# A neoceratopsian dinosaur from the early Cretaceous of Mongolia and the early evolution of ceratopsia

**DOI:** 10.1038/s42003-020-01222-7

**Published:** 2020-09-10

**Authors:** Congyu Yu, Albert Prieto-Marquez, Tsogtbaatar Chinzorig, Zorigt Badamkhatan, Mark Norell

**Affiliations:** 1 Division of Vertebrate Paleontology, American Museum of Natural History, New York, 10024 USA; 2 Institut Català de Paleontologia Miquel Crusafont, ICTA-ICP, Edifici Z, c/de les Columnes s/n Campus de la Universitat Autònoma de Barcelona, 08193 Cerdanyola del Vallès Sabadell, Barcelona, Spain; 3 Department of Biological Sciences, North Carolina State University, Raleigh, NC 27695 USA; 4 Institute of Paleontology, Mongolian Academy of Sciences, Ulaanbaatar, 15160 Mongolia; 5 Department of Earth Sciences, Montana State University, Bozeman, MT 59717 USA

**Keywords:** Palaeontology, Phylogenetics

## Abstract

Ceratopsia is a diverse dinosaur clade from the Middle Jurassic to Late Cretaceous with early diversification in East Asia. However, the phylogeny of basal ceratopsians remains unclear. Here we report a new basal neoceratopsian dinosaur *Beg tse* based on a partial skull from Baruunbayan, Ömnögovi aimag, Mongolia. *Beg* is diagnosed by a unique combination of primitive and derived characters including a primitively deep premaxilla with four premaxillary teeth, a trapezoidal antorbital fossa with a poorly delineated anterior margin, very short dentary with an expanded and shallow groove on lateral surface, the derived presence of a robust jugal having a foramen on its anteromedial surface, and five equally spaced tubercles on the lateral ridge of the surangular. This is to our knowledge the earliest known occurrence of basal neoceratopsian in Mongolia, where this group was previously only known from Late Cretaceous strata. Phylogenetic analysis indicates that it is sister to all other neoceratopsian dinosaurs.

## Abstract (web summary)

Congyu Yu et al. present the partial skull of a new basal neoceratopsian dinosaur from Mongolia. They show that this group is a sister taxon to all other neoceratopsian dinosaurs, and that it occurs earlier in the Cretaceous than previously shown.

## Introduction

During the last three decades, several basal ceratopsians have been unearthed in Upper Jurassic and Lower Cretaceous strata in Asia and North America, including *Yinlong downsi*^1^, *Chaoyangsaurus youngi*^2^, *Xuanhuaceratops niei*^3^, *Liaoceratops yanzigouensis*^4^, *Archaeoceratops oshimai*^5^, *Auroraceratops rugosus*^6^, and *Aquilops americanu*s^7^. However, the phylogenetic relationships among early neoceratopsians and with other ceratopsians are debated. Some hypotheses suggest that Psittacosauridae and Neoceratopsia form a monophyletic clade that is the sister group to other basal ceratopsians, which are paraphyletic^8^. Others have proposed an alternative hypothesis where Chaoyangsauridae (including *Chaoyangsaurus*, *Xuanhuaceratops*, *Hualianceratops*, and *Yinlong*) and *Psittacosaurus* together form a sister group relationship to Neoceratopsia^9^. Although the monophyly of Psittacosauridae and Neoceratopsia is well established, the phylogenetic relationships among ceratopsians remain to be fully resolved. Moreover, there are limited ceratopsian fossil records until the middle Late Cretaceous.

Here we report a new basal neoceratopsian dinosaur, *Beg tse* gen et sp. nov., that was found near the town of Barunnbayan in Mongolia (see supplementary data). This taxon exhibits a diverse and unique combination of characters. Phylogenetic analyses show that *Beg* is the most basal neoceratopsian dinosaur known to date and is more derived than both Psittacosauridae and Chaoyangsauridae. This species also adds the diversity of ceratopsian dinosaurs around the Early/Late Cretaceous.

## Results

### Institutional abbreviations

AMNH-FARB, American Museum of Natural History, collection of fossil amphibians, reptiles and birds, New York, USA; IGM, Institute of Paleontology, Mongolian Academy of Sciences, Ulaanbaatar-15160, Mongolia.

### Material

The holotype and only known specimen of *Beg tse* was collected near the town of Tsogt-Ovoo, Ömnögovi aimag (Province) in Mongolia during the 2015 Mongolian Academy of Sciences-American Museum of Natural History Joint Paleontological Expedition. GPS coordinates are available upon reasonable request from the corresponding author or the archives of the AMNH Vertebrate Paleontology Department. A 3D surface scan of the skull is available from the senior author or the AMNH Vertebrate Paleontology Department. The specimen was CT scanned with available technology, but because of the complex matrix (especially metals) embedded in the specimen, the scan provided little information.

### Systematic Paleontology

Ornithischia Seeley, 1887.

Marginocephalia Sereno, 1986.

Ceratopsia Marsh, 1890.

Neoceratopsia Sereno, 1986

*Beg tse* gen. et sp. nov.

### Etymology

The name is derived from the Himalayan deity Beg-tse. In Mongolian culture, it refers to a pre-Buddhist god of war. Beg-tse is commonly portrayed as heavily armored with large rugosities on its body, which refers to the rugose structures on the jugal and surangular.

### Holotype

IGM 100/3652 is a partial skeleton including an articulated partial skull and extremely fragmentary postcrania. The left side of the skull is best preserved and nearly complete, lacking the rostral, postorbital, squamosal, and most of the parietal. The postcranial elements are represented by a rib, a partial left scapula, a proximal region of the right ischium, and several unidentifiable bone fragments.

### Locality and horizon

The *Beg* type locality is around 14 km from the town of Tsogt-Ovoo. The red beds at this locality used to be considered as outcrops of the Baruunbayan Formation and were first documented by the Soviet-Mongolian Paleontological Expeditions between 1946 and 1949. These beds are now considered to be part of the Ulaanoosh Formation. Shuvalov^10^ recognized the red beds at Baruunbayan as part of the Baruunbayan Svita (svita is a former Soviet chronostratigraphic unit) based on exposures near mountains adjacent to the towns of Baruunbayan and Zuunbayan. Later, the Baruunbayan Svita was more thoroughly studied during a large geological mapping project carried out in the same area by a variety of studies^11,12^. Due to insufficient representation (only partial sections are exposed at these localities), Badamgarav et al.^11^ proposed that the Ulaanoosh Formation represents a complete section of red beds in the Baruunbayan area based on drill logs. The Ulaanoosh Formation is distributed in the areas of Ulaanoosh, Alguu Ulaan Tsav, Baruunbayan, and Zuunbayan^11^. The age of the Ulaanoosh Formation ranges from late Early to early Late Cretaceous (Albian-Cenomanian) based on vertebrate and invertebrate fossils such as dinosaurs, dinosaur eggs, molluscs, ostracods, and turtles^13–16^. The Ulaanoosh Formation is composed of two members: a lower and an upper. The lower member (Aptian-Albian) is comprised of white-colored mudstone, fine-grained conglomerates, bright gray colored, carbonate-rich sandstone, yellowish fine-grained sandstone with carbonate concretions and gray colored conglomerates preserving invertebrate fossils^11,15,17,18^. The upper member is largely composed of reddish colored, fine-medium grained mudstone, fine-medium grained conglomerate, and a breccia layer in the base^11^. The upper member of the formation preserved fragmentary sauropods^14,15^ and dinosaur eggs Faveoloolithus^12^.

Absolute ages and stratigraphic correlation among many Gobi Desert localities are difficult to investigate and hindered by a lack of detailed geological mapping and sediments suitable for radiometric dating. The rock matrix embedding *Beg* is similar to sediments of the lowest upper member of Ulaanoosh Formation in being composed of reddish-brown conglomerate-breccia and sandstones^11^. This suggests the age of this specimen can be constrained between 113~94 Ma, most likely at the boundary between Lower and Upper Cretaceous (~100.5 Ma). Therefore, here we posit that *Beg* is from the latest Early Cretaceous or the earliest Late Cretaceous.

### Diagnosis

IGM 100/3652 is a ceratopsian dinosaur possessing the following autapomorphies: ‘L-shaped’ lacrimal, a small foramen on the medial surface of the anterior third of the jugal anterior ramus, a premaxilla with four enlarged cylindrical teeth, five equally spaced tubercles on lateral surface of surangular, and a large deep pit on the ventral exposure of the jugal. Besides these autopomorphies, its trapezoidal antorbital fossa with poorly delineated rostral margin and shortened dentary with lateral grooves are also different from the basal ceratopsians.

## Description

### General description of the skull

The skull is transversely broad and dorsoventrally low, with a basal length of ~140 mm (as preserved, from the incomplete predentary tip to the surangular, lacking the rostral and squamosal bones). The distance from the left jugal to the mid-line of the skull is 89 mm. These dimensions place the specimen among medium-sized basal ceratopsians^19^.

In lateral view, the skull is anteroposteriorly short and robust, displaying a deep rostrum unlike that of other basal neoceratopsians like *Liaoceratops*^4^ and *Aquilops*^7^. It resembles later neoceratopsian species, such as *Auroraceratops*^20^, in having a dorsoventrally deep premaxilla, for example. The orbit and antorbital fossae are relatively large with diameters of 47 and 25 mm, respectively. This is in contrast to the comparatively reduced naris with a diameter of only 14 mm. The nasal opening is dorsoventrally deep, elliptical and whose ventral and posterior sides are well defined by the left premaxilla on the left side. The nasal passage is filled with the unpreparable matrix. The anterior region of the braincase is preserved, while the posterior elements and the parietal, squamosals, and most of the postorbitals are missing. The left half of the anterior region of the skull is relatively complete, including a partial premaxilla, partial nasal, maxilla, fragmentary predentary, and dentary.

The antorbital fossa is subtrapezoidal and the anterior margin is poorly delineated near the articulation between the premaxilla and maxilla. The lacrimal contributes mostly to the dorsal margin of antorbital fossa and the posterior border is mainly formed by the anterior edge of the jugal. The dorsal margin of the lacrimal forms a sharp anteriorly descending crest. This, in combination with the jugal, contributes to the formation of the antorbital foramen that lies posterior to the fossa. The antorbital fenestra lies within the antorbital fossa and extends anteriorly. It is hidden by the jugal from lateral exposure. The antorbital fenestra connects the orbital cavity and antorbital fossa. In basal ceratopsians, the size and shape of the antorbital fenestra vary a lot. While *Archaeoceratops* has a large triangular antorbital fossa^5^, *Yinlong*^1^, *Liaoceratops*^4^, and *Psittacosaurus*^21^ have antorbital fossae that are reduced with gradual boundaries. The subtrapezoidal shape of antorbital fossa in *Beg* is unique among basal ceratopsians.

Only the anterior, ventral, and partially dorsal margins of the orbit are preserved. These margins are composed of the lacrimal, and the prefrontal and jugal, respectively. The partially preserved frontal roughly forms the outline of the dorsal part of the orbital rim and a horn-like lateral projection is present in dorsal view. Unlike *Auroraceratops*^6^ or *Hualianceratops*^9^, the jugal forms only a slightly concave margin of the ventral orbital rim, rather than a curved border. Only a small portion of the postorbital is preserved adjacent to the dorsal-most region of the jugal, where there is a remarkably deep groove. This groove may be an articulation for postorbital or accommodate its missing portion overlapping the jugal. Only part of the left frontal is preserved, albeit slightly distorted. The level of the orbit is dorsal to both the external naris and the antorbital fossa while the ventral-most rim lies lower than the dorsal-most border of the external naris and antorbital fossa. This, however, could be affected by postmortem deformation.

The anterior border of the temporal fenestra is formed by the posterior margin of the jugal. The fenestra is superficially small because the quadrate was crushed into a position slightly more posterior than the posterior margin of the jugal. Although the parietal and squamosal are not preserved in this specimen, the proportions of the skull show that the supratemporal fenestra was probably much smaller than the orbit, as in *Auroraceratops*^6^ and *Liaoceratops*^4^.

### Premaxilla and premaxillary teeth

Unlike other basal neoceratopsians, the premaxilla of *Beg* is deep and trapezoidal in lateral view, resembling that of *Psittacosaurus* and derived neoceratopsians^19^. There is a projection on its anterior edge, above which is the external naris. All ceratopsian dinosaurs have a unique rostral bone^19^. Although not preserved, the recess on the rostroventral region of the premaxilla strongly indicates the presence of an articulation surface for a small rostral bone similar to those of other basal neoceratopsians. The posterolateral process of the premaxilla contacts both the lacrimal and prefrontal, excluding the maxilla from nasal contact. Both contacts are dorsoventrally wide, covering almost the entire anterior margins of the prefrontal and lacrimal. A shallow horizontal groove is present in the middle part of the premaxilla, forming a depression at the premaxilla-maxilla boundary. Only a small portion of the nasal is preserved in the holotype. The preserved part forms a smooth anteriorly descending surface with the prefrontal. As in other basal ceratopsians, there is no sign of a nasal horn^4,7,22^.

The lack of the anterior and ventral parts of the premaxilla allows for the roots of the premaxillary teeth to be observed on the left lateral side. These teeth are slightly recurved and cylindrical in cross section. The second premaxillary tooth is the most complete, the exposed tooth measuring 11 mm in length. The third premaxillary tooth has erupted, and the first and last are incompletely preserved. Premaxillary teeth are common among basal ceratopsians except psittacosaurs. However, *Beg* is the only known taxon with four premaxillary teeth while other basal ceratopsians have three (*Yinlong*, *Aquilops*, *Liaoceratops*, *Archaeoceratops*, and *Auroraceratops*) or fewer^1,4,6,7^.

### Lacrimal

The lacrimal is shaped like a tilted inverted ‘L’. Other basal ceratopsians, such as *Yinlong, Liaoceratops*, and *Aquilops*, lack the anterior ramus^1,4,7^. A palpebral bone is apparently missing in *Beg*. The anterior and posterior rami of the lacrimal are nearly equal in length, but the former is thinner. The anterior end of the lacrimal contacts the premaxilla, and a rostroventral process joins both the premaxilla and maxilla, overlapping their sutures. The posteroventral end of the lacrimal tapers toward the anterodorsal end of the jugal, which forms the anterior and dorsal margins of the orbit. In lateral view, the middle section of the lacrimal is transversely thick, forming a nearly right angle with the antorbital fossa. et al.

### Frontal

Nearly the entire frontal is exposed due to the unpreserved adjacent elements, allowing the connection of the nasal passage and the external naris to be observed. The frontal is transversely broad in dorsal view, dorsoventrally thin near the edges and thick near the midline. Also, in the dorsal view, there is a notable spike projecting posteriorly from the lateral edge of left frontal. Though most ornithischian dinosaurs are characterized by a palpebral bone on the anterodorsal margin of the orbit, this spike is part of the frontal and covers the posterior half of the orbit, and therefore, it cannot be palpebral, unless it is palpebral that has fused to the frontal. The spike on the right-side is not preserved. Though such lateral projection has not been observed in other ornithischian dinosaurs, we cannot rule out the possibility that was formed by fracture, therefore we do not take this feature as an autopomorphy.

### Maxilla and maxillary teeth

The maxilla is dorsoventrally deep and may be divided into upper and lower regions by a posteriorly expanded crest, which is similar to the deep emargination of *Hualiaceratops*^9^. This crest forms the ventral margin of the large antorbital fossa. The dorsal border of the maxilla is overlapped by the lacrimal and jugal and the anterior region is capped by the dorsal border of the antorbital fossa. A row of at least six neurovascular foramina are present ventrally on the left maxilla, and four similar foramina occur on the right counterpart. Maxillary teeth are mostly embedded in the matrix yet resemble those of other basal ceratopsians in that they display several ridges on their buccal surfaces^1,8^. However, unlike the more derived *Protoceratops* and ceratopsids, a prominent ridge on the buccal surface of the maxillary teeth in *Beg* is lacking. There are three teeth exposed on the left maxilla and at least six on the right maxilla. Considering the available space, *Beg* probably possessed fewer cheek teeth than most neoceratopsians. It seems that *Beg* has fewer teeth than one of the most basal neoceratopsian *Liaoceratops*, which has at least eight maxillary teeth in juvenile specimen IVPP V12633 and 13 in adult specimen IVPP V12738^4^. This is further suggested by the sparse arrangement of the preserved teeth, a plesiomorphic condition for ceratopsians^23^. The tooth row is relatively deep in lateral view and lies ventral to the orbit. The upper and lower dentitions are tightly appressed, concealing the morphology of their crowns and occlusal surfaces.

### Jugal

The anterior terminus of the jugal resembles that of *Psittacosaurus*^21,24^ in having a blunt surface that contributes substantially to the antorbital fossa. The jugal expands posteriorly to conceal much of the quadratojugal, unlike in basal ceratopsians such as *Chaoyangsaurus*^2^ and *Yinlong*^1^. There is a horizontal ridge along the posterior third of the jugal. Other basal ceratopsians either do not have this structure or have a tilted ridge ventroposteriorly, for example, *Psittacosaurus* and juvenile *Liaoceratops*^4,21^. However, there is no pronounced jugal boss, a conspicuous condition present in many ceratopsians including *Psittacosaurus*^24^*. Beg* also lacks an epijugal bone, a diagnostic character of more derived taxa^19^. Notably, the anterior and central regions of the lateral surface of the jugal bear a series of tubercles. In contrast, the posterior region is relatively smooth. Similar sculpturing is present in the basal ceratopsian *Yinlong*^1^ and *Hualianceratops*^9^. Anteriorly, there is a foramen on the medial surface of both jugals, near their contact with the maxilla. On the ventral surface of the left jugal, there is a deep groove that opens anteriorly at the posterior corner of the antorbital fossa. Such a groove is not present in any other ceratopsian, or at least to our knowledge, any available specimens such as the *Protoceratops*^25^, or *Yinlong*^1^.

### Quadrate

The quadrate is partially hidden in between the posterior third of the jugal and the braincase, so that only its dorsal and posterior extents are exposed. The quadrate displays a nearly vertical posterior ridge that bifurcates dorsally to form an inverted triangular quadrate foramen. The vertical ridge on the occipital surface of quadrate is unlike most other ceratopsian dinosaurs, for example, *Yinlong* and *Protoceratops* which have a relatively smooth occipital surface^1,25^. When articulated, the quadrate would have lain more posteriorly, providing sufficient space to accommodate the quadratojugal and pterygoid. A shallow fossa exists on the middle of the lateral surface of the quadrate that is similar to *Liaoceratops*^4^, slightly above the quadratojugal contact. The quadrate-quadratojugal joint is not observable, due to the distortion of the posterior part of the skull.

### Braincase

The distorted braincase has been taphonomically pushed laterally to the right side of the skull relative to the parasagittal axis, as well as shifted anteriorly. The relative positions of the bones are, however, still intact. The foramen magnum and occipital condyle are not visible as they are embedded in the unpreparable matrix. Yet, both exoccipitals are preserved in articulation with their lateral regions exposed. The basioccipital and supraoccipital are also embedded in a matrix, preventing determination as to whether the supraoccipital contributes to the dorsal margin of the foramen magnum. Although the fusion of exoccipitals and opisthotics are widely observed in ornithischian dinosaurs^26,27^, the opisthotic contribution cannot be clearly observed as there is no definitive exposure from the occipital view. There is a horizontal ridge on the posterior surface of exoccipital separating it into equally sized upper and lower parts. Due to deformation, the left paroccipital process is tightly fused to the left quadrate, preventing observation of additional neurocranial elements. Several openings are observed around the junction of the occipital elements. Using *Protoceratops andrewsi* as ref. ^25^, these openings can be attributed to cranial nerves IX to XII. In *Protoceratops*, cranial nerves IX to XI share a single opening and cranial nerve XII may have exited through more than one. Openings for the other cranial nerves (which might have been oriented laterally or anteriorly) and the occipital condyle are embedded in the matrix.

### Mandible

Except for the predentary, most of the left mandible is well preserved. The mandible is anteroposteriorly short and dorsoventrally deep in lateral view, with a slightly convex ventral profile. The overall morphology resembles other basal ceratopsian such as *Yinlong* and *Liaoceratops*^1,4,28^, but its curvature is less significant than derived taxa like *Protoceratops*^25^. The dentary, surangular, and angular are tightly articulated with hardly any visible joints. The whole mandible is mediolaterally expanded but the tooth rows are arranged parasagittally, leaving wide buccal regions. The posterior half of the mandible is laterally expanded with a robust coronoid process. There is no sign of an external mandibular fenestra as in *Yinlong*, which may be the condition in other basal ceratopsians^1^. A shallow and wide groove connects the middle region of dentary with both the surangular and the angular, which is shallower and more elongated than the fossa on the external surface of the mandible in *Liaoceratops*^4^.

A fragmentary predentary caps the anterior end of the dentary. Although neither its anterior nor posterior margins are clearly recognizable, the preserved parts of the predentary are anteroposteriorly longer than the premaxilla. The presence of a ventral process of the predentary is suggested by a shallow groove extending posteriorly onto the ventral surface of the dentary, where such a process would have articulated. As in most basal ceratopsians, the predentary of *Beg tse* is slender in comparison with that of psittacosaurs and derived ceratopsids^1,24^.

**Fig. 1:**
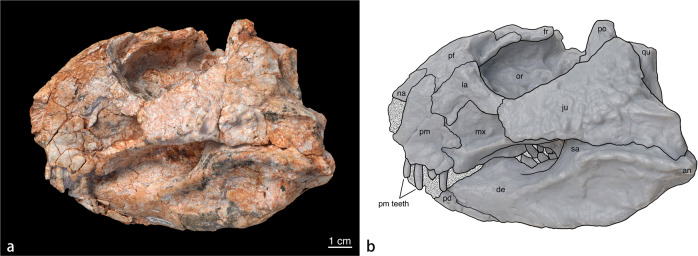
IGM 100/3652. **a** Skull in lateral view. **b** Schematic drawing of skull in left lateral view. Abbreviations: af, antorbital fossa; an, angular; de, dentary; fr, frontal; j, jugal; la, lacrimal; mx, maxilla; na, nasal; or, orbit; pd, predentary; pf, prefrontal; pm, premaxilla; po, postorbital; qu, quadrate; sa, surangular.

**Fig. 2:**
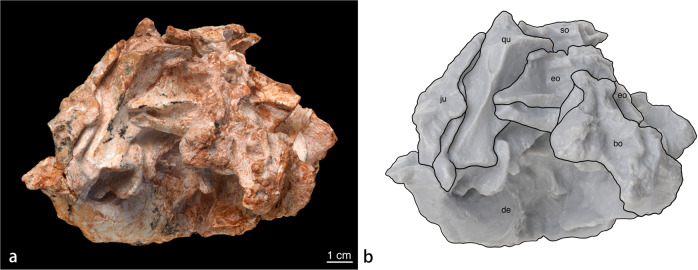
IGM 100/3652. **a** Skull in occipital view. **b** Schematic drawing of the skull in occipital view. Abbreviations: bo, basioccipital; eo, exoccipital; de, dentary; ju, jugal; qu, quadrate; or, orbit; so, supraoccipital.

The dentary is short, slightly curved, and robust. It attains its dorsoventral maximum thickness near the triple junction of the dentary, angular, and surangular. The lateral dentary surface is dorsoventrally concave. The coronoid process is laterally thick and only visible on the less complete right side of the skull. The anterior end of the dentary tooth row is slightly downturned and the dentary tooth row is shorter than that of the maxilla. Most basal ceratopsian have more dentary teeth than maxillary teeth, for example, *Yinlong* and *Liaoceratops*^1,4,28^ The crowns and buccal surfaces of most dentary teeth show signs of extensive wear. Dentary teeth are substantially shorter dorsoventrally than maxillary teeth and lack a clear mesial ridge. Dentary teeth also appear to be more tightly packed than maxillary teeth. Most basal ornithischians have leaf-shaped cheek teeth and only have serrations near the most crown terminus. Derived ceratopsians, for example *Triceratops*, have a prominent central ridge to form the high-angled slicing dentitions^29^. The presence of relatively a strong middle ridge alongside several smaller ridges in cheek teeth of *Beg tse* may be indicative of an intermediate morphological stage (Figs. 1 and 2).

The surangular displays a pronounced lateral ridge bearing five equally spaced tubercles, a condition to our knowledge never observed in any other ceratopsian or dinosaur. Generally, this area of the dentary is smooth^1,4^. The lateral ridge of the surangular is expanded laterally from the jugal surangular contact. The angular is tightly fused with the dentary and surangular, and no articulation is visible. The ventral margin of the angular expands caudodorsally forming a convex edge along the posterior extent of the mandible. However, the lateral surface of the rest of the mandible lacks the textured sculpting observed in many other basal ceratopsians like *Hualianceratops*^9^ and *Yinlong*^1^.

### Phylogenetic analysis

**Fig. 3:**
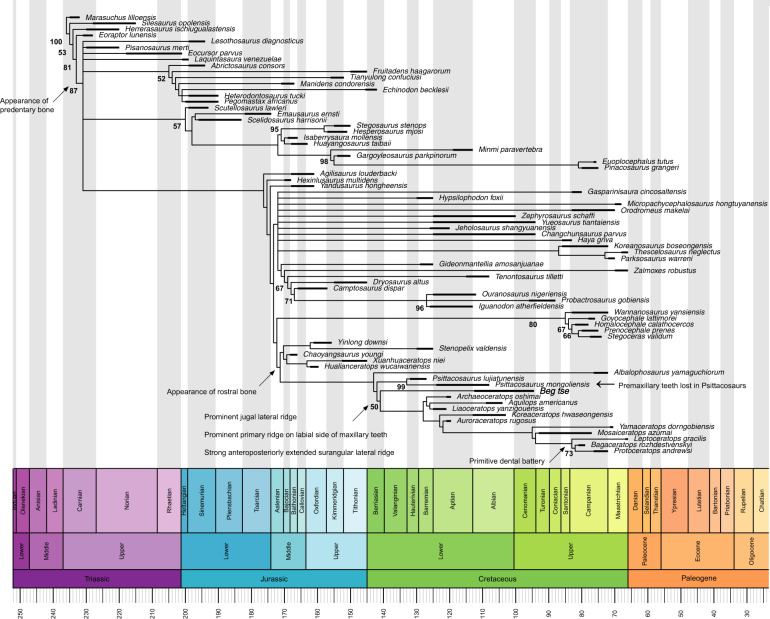
Strict consensus tree of Ornithischia showing the position of *Beg tse*. Created using the R package Strap^37^. The character matrix is from ref. ^35^. Values below nodes represent bootstrap proportions. Arrows indicate key transitional features at nodes.

Our analysis at the ornithischian level resulted in six most parsimonious trees (MPTs, tree length = 1213 steps, CI = 0.369, and RI = 0.71). Using TBR branch swapping in the second round, 30 MPTs were retained. The strict consensus tree shows *Beg* as the most basal neoceratopsian dinosaur (Fig. 3). Psittacosauridae is recovered as sister to Neoceratopsia, followed by the unusual *Albalophosaurus* as sister to the group of Psittacosauridae+Neoceratopsia. A well-defined, discrete Chaoyangsauridae (including *Yinlong*, *Chaoyangsaurus*, *Xuanhuaceratops*, *Hualianceratops*, and *Stenopelix*) was recovered. A fully resolved monophyletic Ceratopsia was also recovered.

**Fig. 4:**
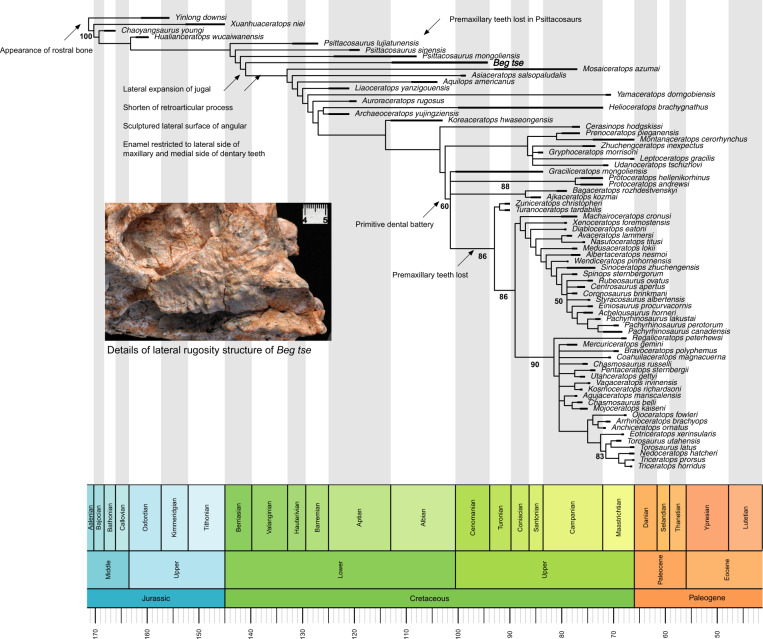
Strict consensus tree of Ceratopsia showing the position of *Beg tse*. Created using the R package Strap^37^. The character matrix is from ref. ^36^. Values below nodes represent bootstrap proportions. Arrows indicate key transitional features at nodes. The inset features details of lateral rugosity structure of *Beg tse*.

In the context of Ceratopsia, the analysis returned five most parsimonious trees (tree length = 747 steps, CI = 0.518, and RI = 0.844). Using TBR branch swapping in the second round, 60 MPTs were retained. *Beg tse* was also recovered to be the sister taxon to all previously described neoceratopsians within the strict consensus tree (Fig. 4). The introduction of *Beg* led to the collapse of Chaoyangsauridae and Psittacosauridae, while Neoceratopsia retained its monophyly.

## Discussion

Like the rugose surface on upper and lower jaws in both *Psittacosaurus* and *Triceratops*, which has been interpreted as attachment points for keratinous structures^21,30^, *Beg* displays a large facial area covered with similar rugosities. Two of the oldest reported ceratopsians, *Yinlong* and *Hualianceratops* have similar rugose structures distributed on their jugals and lower jaws. However, in more derived taxa, with the development of more significant cranial projections including the orbital and nasal horns, jugal bosses, facial elements tend to reduce rugose bony textures. *Yinlong* and *Liaoceratops* display similar tubercles, but these are on posteroventral surface of the surangular in the former and the ventral margin of the angular in the latter. The widespread distribution of tubercles and rugose facial texturing among basal ceratopsians suggest that these might represent a key innovation in early ceratopsian evolution. Other autapomorphies of *Beg* include the foramen on the rostral region of the jugal, the equally spaced tubercles on the lateral ridge of the surangular, the L-shaped lacrimal, and the presence of four premaxillary teeth. As a phylogenetic intermediate between basal ceratopsians and other neoceratopsians, *Beg* also bears several transitional features including relatively tightly arranged teeth without prominent ridges and a laterally expanded flange on the dentary. Although the only informative element preserved is the skull, we can estimate the body size of *Beg* by comparing it with other basal taxa. The 140-mm long skull, though artificially shortened by poor preservation, is comparable in size to adult *Yinlong* and *Liaoceratops* crania. To further investigate the evolutionary changes among early ceratopsians, we mapped the autapomorphies of *Beg tse* and synapomorphies of Neoceratopsia in the ceratopsian and ornithischian trees. The mapping from the strict consensus ceratopsian tree shows all autapomorphies of *Beg tse* are either related to predentary (Character 238 and 239) or teeth (Character 278, 282, and 285). The synapomorphies of Neoceratopsia (*Beg* as the most basal taxon) are from angular (Character 260 and 261), surangular (Character 263), articular (Character 271), and tooth enamel (Character 276). For the ornithischian tree, the autapomorphies of *Beg tse* include the jugal posterior ramus (Character 64), angular tubercle row (Character 194), premaxillary teeth number (Character 196), and cheek tooth primary ridge (Character 211). Neoceratopsia is supported by synapomorphies related to nasal (Character 28, 29), posteroventral dermal plate (Character 152), surangular (Character 182 and 184), and maxillary teeth (Character 209). Both mappings emphasize the importance of posterior elements of the skull and teeth, which are highly related to two key innovations in derived ceratopsians, the expanded neck frill and dental battery.

The oldest known ceratopsian is *Chaoyangsaurus*, which dates back to the Middle Jurassic, possibly Bajocian in age^2^. Other early ceratopsians, including *Yinlong*, *Hualianceratops*, and *Xuanhuaceratops*, are all dated earlier than the J/K boundary^1,3,9^. We disregard *Stenopelix* and *Albalophosaurus* because of their highly ambiguous phylogenetic status, often being placed outside Ceratopsia^31,32^. Neoceratopsians, however, only appear in the fossil record after the Hauterivian (Early Cretaceous, ~130 Ma) but with extremely limited fossil records until the middle Late Cretaceous. The reported oldest taxon *Liaoceratops* was discovered from the lower part of the Yixian Formation^1^. The earliest North American species, *Aquilops americanus*, dates back to the Albian in the late Early Cretaceous^7^. The known diversity of Ceratopsia is significantly lower in the Early and early Late Cretaceous compared to its high diversity in the Campanian and Maastrichtian stages in the Late Cretaceous. A variety of derived features in Ceratopsia, including the expanded neck frill, increased body size, loss of premaxillary teeth, began to emerge in the Early Cretaceous. However, *Beg* still retained the basal state of these characters, suggesting the basic ceratopsian bodyplan was preserved at least until the Early-Late Cretaceous boundary. Taking *Beg* as well as other basal neoceratopsians such as *Mosaiceratops*, *Heliceratops*, and *Auroraceratops*^6,8,33^ into account, they all represent the transitional stage between basal ceratopsians and derived ceratopsids. With a wide geographic range from southern China to Mongolia and long time span from the Aptian to possibly Campanian^33^, the early evolutionary history of basal ceratopsia is more complex than previously thought.

## Methods

### Nomenclatural acts

This published work and the nomenclatural acts it contains have been registered in ZooBank, the proposed online registration system for the International Code of Zoological Nomenclature (ICZN). The ZooBank LSIDs (Life Science Identifiers) can be resolved and the associated information viewed through any standard web browser by appending the LSID to the prefix “http://zoobank.org/”. The LSID for this publication is: D5EDF B08-46FC-4D1B-9D3F-F5B60B3DCE7A.

### Statistics and reproducibility

The analyses were conducted using the new technology search algorithm in TNT version 1.1^34^. Parameters were left in the default setting. The phylogenetic position of *Beg* within both Ornithischia and Ceratopsia was inferred via parsimony. We used two updated data matrices modified from Han et al.^35^ and Knapp et al.^36^ (see supplementary data), containing 73 species with 380 unordered characters and 71 species with 350 unordered characters, respectively.

### Reporting summary

Further information on research design is available in the Nature Research Reporting Summary linked to this article.

## Supplementary information

Supplementary Information

Description of Additional Supplementary Files

Supplementary Data

Reporting Summary

## Data Availability

The authors declare that the data supporting the findings of this study are available in the paper and its supplementary information files. The specimen is held in IGM with a specimen number of IGM-100/3652.
