# Supplementary material for: A neoceratopsian dinosaur from the early Cretaceous of Mongolia and the early evolution of ceratopsia
Source: Commun Biol. 2020 Sep 10;3:499. doi: 10.1038/s42003-020-01222-7 (PMC7484756; doi:10.1038/s42003-020-01222-7)
Supplement: Supplementary file 1 — Supplementary Information [file 42003_2020_1222_MOESM1_ESM.pdf]

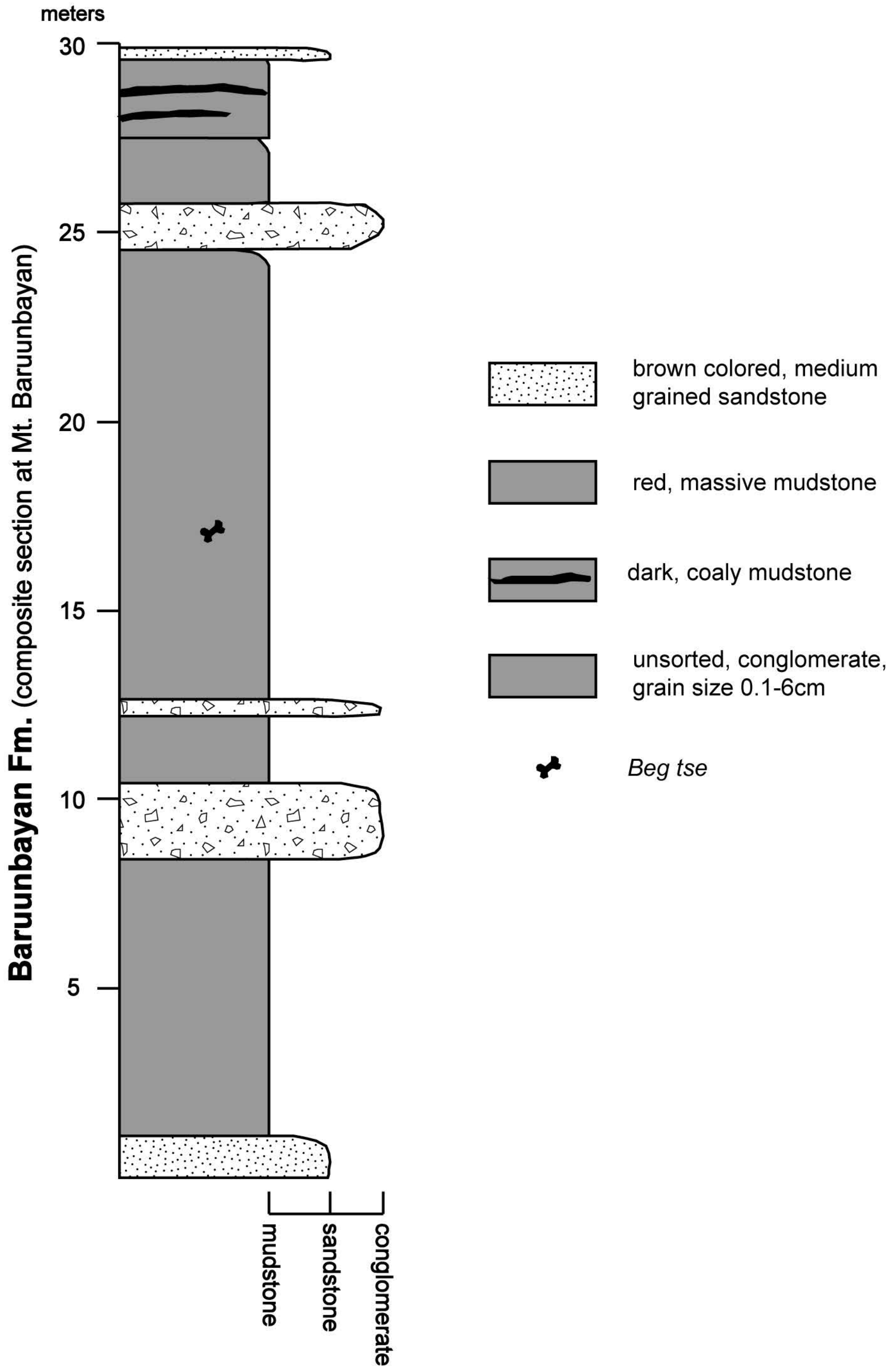

Supplementary Figure 1. Stratigraphic section at Baruunbayan, Mongolia

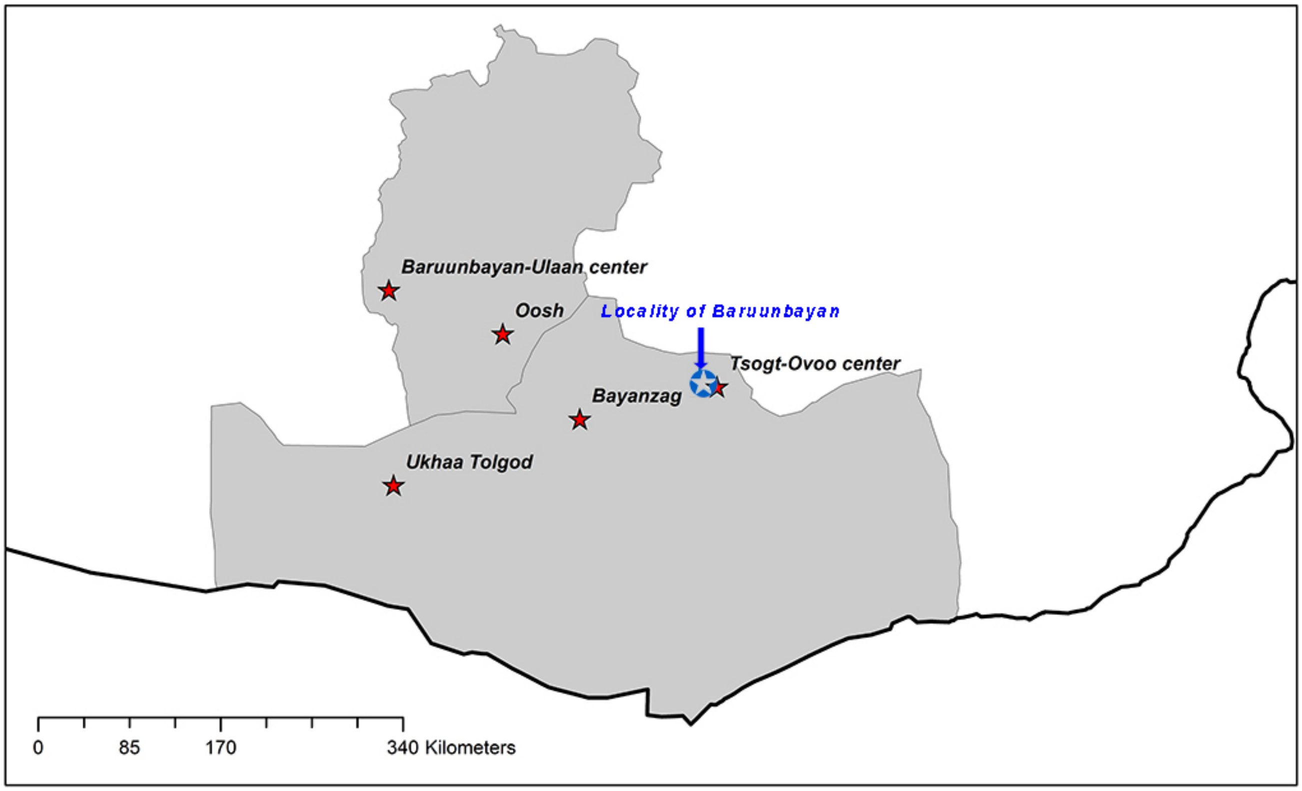

Supplementary Figure 2. Fossil locality of *Beg tse*
