# Supplementary material for: A neoceratopsian dinosaur from the early Cretaceous of Mongolia and the early evolution of ceratopsia
Source: Commun Biol. 2020 Sep 10;3:499. doi: 10.1038/s42003-020-01222-7 (PMC7484756; doi:10.1038/s42003-020-01222-7)
Supplement: Supplementary file 2 — Description of Additional Supplementary Files [file 42003_2020_1222_MOESM2_ESM.docx]

**Description of Additional Supplementary Files**

**Supplementary Data:** Panel 1. Ornithischian level character matrices of *Beg tse* for software TNT. Panel 2. Ceratopsian level character matrix of *Beg tse* for software TNT.
