# Supplementary material for: A neoceratopsian dinosaur from the early Cretaceous of Mongolia and the early evolution of ceratopsia
Source: Commun Biol. 2020 Sep 10;3:499. doi: 10.1038/s42003-020-01222-7 (PMC7484756; doi:10.1038/s42003-020-01222-7)
Supplement: Supplementary file 4 — Reporting Summary [file 42003_2020_1222_MOESM4_ESM.pdf]

nature research

Corresponding author(s): Congyu Yu

Last updated by author(s): Jun 21, 2020

Reporting Summary

Nature Research wishes to improve the reproducibility of the work that we publish. This form provides structure for consistency and transparency in reporting. For further information on Nature Research policies, see our Editorial Policies and the Editorial Policy Checklist.

Statistics

For all statistical analyses, confirm that the following items are present in the figure legend, table legend, main text, or Methods section.

n/a	Confirmed	
☐	☒	The exact sample size (*n*) for each experimental group/condition, given as a discrete number and unit of measurement
☒	☐	A statement on whether measurements were taken from distinct samples or whether the same sample was measured repeatedly
☒	☐	The statistical test(s) used AND whether they are one- or two-sided<br>*Only common tests should be described solely by name; describe more complex techniques in the Methods section.*
☒	☐	A description of all covariates tested
☒	☐	A description of any assumptions or corrections, such as tests of normality and adjustment for multiple comparisons
☒	☐	A full description of the statistical parameters including central tendency (e.g. means) or other basic estimates (e.g. regression coefficient) AND variation (e.g. standard deviation) or associated estimates of uncertainty (e.g. confidence intervals)
☒	☐	For null hypothesis testing, the test statistic (e.g. F, t, r) with confidence intervals, effect sizes, degrees of freedom and P value noted<br>*Give P values as exact values whenever suitable.*
☒	☐	For Bayesian analysis, information on the choice of priors and Markov chain Monte Carlo settings
☒	☐	For hierarchical and complex designs, identification of the appropriate level for tests and full reporting of outcomes
☒	☐	Estimates of effect sizes (e.g. Cohen's d, Pearson's r), indicating how they were calculated

Our web collection on statistics for biologists contains articles on many of the points above.

Software and code

Policy information about availability of computer code

Data collection	Adobe Photoshop CC 2019 and Adobe Illustrator CC 2019 are used for imaging processing and schematic drawing.
Data analysis	Software TNT 1.1 (Goloboff et al., 2008, Cladistics) is used in this study for phylogenetic analysis,<br>Strap package for R (Bell and Lloyd, 2014) is used in Rstudio 3.5.1 for image plotting.

For manuscripts utilizing custom algorithms or software that are central to the research but not yet described in published literature, software must be made available to editors and reviewers. We strongly encourage code deposition in a community repository (e.g. GitHub). See the Nature Research guidelines for submitting code & software for further information.

Data

Policy information about availability of data

All manuscripts must include a data availability statement. This statement should provide the following information, where applicable:

- Accession codes, unique identifiers, or web links for publicly available datasets
- A list of figures that have associated raw data
- A description of any restrictions on data availability

The authors declare that phylogenetic data supporting the findings of this study are available within the paper and its supplementary information files. The geological coordinates of fossil sites are available from the corresponding author or the Division of Paleontology in American Museum of Natural History upon reasonable request.

Field-specific reporting

Please select the one below that is the best fit for your research. If you are not sure, read the appropriate sections before making your selection.

☐ Life sciences ☐ Behavioural & social sciences ☒ Ecological, evolutionary & environmental sciences

For a reference copy of the document with all sections, see nature.com/documents/nr-reporting-summary-flat.pdf

Ecological, evolutionary & environmental sciences study design

All studies must disclose on these points even when the disclosure is negative.

Study description	We described a ceratopsian specimen (IGM 100/3652) based on a partial skull and reconstruct its phylogenetic status at both ceratopsian and ornithischian levels.
Research sample	Specimen described: IGM 100/3652, a partial ceratopsian skull discovered in southern Mongolia<br>Phylogenetic analysis at ceratopsian level: 73 species and 380 unordered characters, data from Knapp et al., (2018)<br>Phylogenetic analysis at ornithischian level: 71 species and 350 unordered characters, data from Han et al., (2017)
Sampling strategy	Only sample available in the study was the partial skull IGM 100/3652
Data collection	Character coding of specimen were followed Knapp et al. (2018) for ceratopsian phylogeny and Han et al. (2017)
Timing and spatial scale	The specimen was collected near Tosgt-Ovoo center, Ömnögovi aimag in Mongolia during the 2015 Mongolian Academy of Sciences-American Museum of Natural History Joint Paleontological Expedition.
Data exclusions	N/A
Reproducibility	N/A
Randomization	N/A
Blinding	N/A

Did the study involve field work? ☐ Yes ☒ No

Reporting for specific materials, systems and methods

We require information from authors about some types of materials, experimental systems and methods used in many studies. Here, indicate whether each material, system or method listed is relevant to your study. If you are not sure if a list item applies to your research, read the appropriate section before selecting a response.

Materials & experimental systems

n/a	Involved in the study	
☒	☐	Antibodies
☒	☐	Eukaryotic cell lines
☐	☒	Palaeontology and archaeology
☒	☐	Animals and other organisms
☒	☐	Human research participants
☒	☐	Clinical data
☒	☐	Dual use research of concern

Methods

n/a	Involved in the study	
☒	☐	ChIP-seq
☒	☐	Flow cytometry
☒	☐	MRI-based neuroimaging

Palaeontology and Archaeology

Specimen provenance	The permits were issued by Ministry of Education, Culture, and Science (Mongolia) in 25th, June, 2015
Specimen deposition	the specimens have been deposited at the Institute of Paleontology and Geology, Mongolian Academy of Sciences (IPG-MAS)
Dating methods	N/A, dating of this specimen was according to previous studies. Details are in Horizon and locality section in the manuscript.

☒ Tick this box to confirm that the raw and calibrated dates are available in the paper or in Supplementary Information.

Ethics oversight	There is no ethical approval or guidance required for this study.

Note that full information on the approval of the study protocol must also be provided in the manuscript.
